# Biobased Random Copolymers of Poly(Hexamethylene Furanoate) for Sustainable Food Packaging: Camphoric Acid as a Valuable Co-Monomer for Improved Mechanical Properties

**DOI:** 10.3390/polym18020255

**Published:** 2026-01-17

**Authors:** Enrico Bianchi, Michelina Soccio, Valentina Siracusa, Massimo Gazzano, Nadia Lotti

**Affiliations:** 1 Department of Civil, Chemical, Environmental and Materials Engineering, University of Bologna, Via Terracini 28, 40131 Bologna, Italy; enricobianchi1994@gmail.com (E.B.); m.soccio@unibo.it (M.S.); 2 Interdepartmental Center for Industrial Research on Advanced Applications in Mechanical Engineering and Materials Technology, CIRI-MAM, University of Bologna, Via Ugo Foscolo 7, 40123 Bologna, Italy; 3 Department of Chemical Science, University of Catania, Viale A. Doria 6, 95125 Catania, Italy; vsiracus@dmfci.unict.it; 4 Organic Synthesis and Photoreactivity Institute, ISOF-CNR, Via Gobetti 101, 40129 Bologna, Italy; massimo.gazzano@cnr.it; 5 Interdepartmental Center for Agro-Food Research, CIRI-AGRO, University of Bologna, Via Quinto Bucci 336, 47521 Cesena, Italy

**Keywords:** 2,5-FDCA, mechanical properties, gas permeability properties, random copolymer, polymerization, flexible packaging, compression molding

## Abstract

In recent years, the unsustainable consumption of fossil resources has been causing major ecological concerns, especially for the production of polymeric materials. 2,5-furandicarboxylic acid (FDCA) is one of the most appealing biobased chemical building blocks, because of its potential to replace the industrially widespread petrochemical, terephthalic acid. Camphoric acid (CA) is also an interesting biobased chemical derived from camphor, one of the most widespread fragrances. This work had the objective of combining CA, FDCA and biobased 1,6-hexanediol to synthesize random copolymers for sustainable food packaging applications by means of a solvent-free polycondensation process, obtaining poly(hexamethylene furanoate-co-camphorate)s (PHFC). The optimization of the synthesis made it possible to obtain high molecular weight polyesters with a percentage of camphoric acid up to 17 mol%, which could be compression-molded into films. They were subjected to molecular, structural, thermal and functional characterization via NMR, GPC, WAXS, DSC, and TGA analyses, as well as mechanical and gas permeability tests. Compared to the homopolymer of reference, it was possible to obtain higher flexibility, 430% higher elongation at break, and 223% higher toughness, with comparable, excellent gas permeability properties. Calorimetric evidence suggested that camphoric acid might have enhanced the formation of a partially ordered mesomorph phase in the copolymers under study.

## 1. Introduction

**Figure 1:**
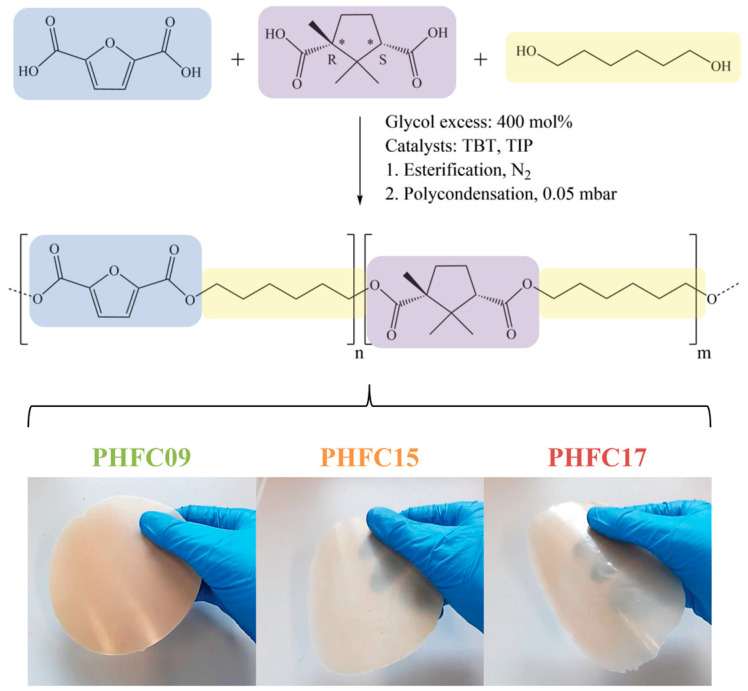
Reaction scheme and compression-molded films of PHFC09, PHFC15, and PHFC17. * identifies a chiral center.

The use of plastics has undeniably become an essential component of contemporary society, with their manufacturing, utilization, and disposal posing increasing ecological challenges. The accumulation of plastic waste in various environments, including oceans and rivers, has become concerning due to the persistence and resistance to degradation of most petrochemical commodities. These issues lead to an increasing interest, both legislative [1,2] and academic [3,4,5,6], in the circularity of plastics, from their production to their end-of-life. In particular, as part of the solution, biobased polymers are a highly relevant field of research: the substitution of petrochemical plastics with biobased ones has been shown, through LCA studies to lead to a relevant decrease in CO_2_ emissions [7], paired with minimal employment of agricultural land for dedicated crops [8], or even with the utilization of agricultural waste in extremely high added value processes [9]. Moreover, renewable resources are less exposed to the fluctuations in price typical of fossil feedstock, and are more accessible because of the wide availability of biomass on the planet. Packaging is the main market application of polymeric materials, amounting to 39% of the 54.6 million tons of plastics produced in 2024 in Europe. Within this field of application, it is known that flexibility can be a key aspect to reduce the carbon footprint of food packaging. In fact, flexible packaging has a packaging-to-product ratio 5 to 10 times lower than rigid packaging, and for this reason, packing all food in flexible packaging has been projected to have the potential to reduce the total carbon emissions for packaging by 40% in Europe [10]. In this context, 2,5-furandicarboxylic acid is one of the most interesting biobased building blocks [11,12], with a great potential to be used as monomer for the production of renewable polyesters [13,14,15,16,17], such as poly(ethylene furanoate) (PEF) and poly(propylene furanoate) (PPF). In general, polyesters from 2,5-FDCA have proven to reach exceptional functional properties, especially in the field of food packaging [18,19,20]. In particular, PEF and PPF can be obtained from the polymerization of 2,5-FDCA with ethylene glycol and propylene glycol, respectively, and they have been drawing the attention of industry [15,21,22] in recent years: in fact, PEF will be introduced into the market in 2024, commercialized for the production of biobased bottles [23]. However, the costs for the production of biobased 2,5-FDCA are still a factor preventing the industrialization of FDCA-based chemicals and polymers at a truly competitive price, compared to petrochemical alternatives. In fact, biobased 2,5-FDCA is currently obtained from first gen biomass such as starch, glucose, or high-fructose corn syrup [24,25]: by means of techno-economic analyses, it was possible to evaluate low-cost and high-efficiency technologies for the production of 2,5-FDCA from this feedstock, which resulted in a minimum selling price of 1.80 USD kg^−1^ [25], making 2,5-FDCA more than twice as expensive to produce as terephthalic acid (TA), which is currently sold at 0.86 USD kg^−1^ [26] and is the monomer used for the production of poly(ethylene terephthalate) (PET), the petrochemical alternative to PEF. In the case of first gen PEF, it is expected that the market price at an industrial scale (100 kton per year) will be between 4.62 and 5.77 USD kg^−1^ [24], making the polymer about four times as expensive as PET, currently sold at 1.24 USD kg^−1^ [27]. Similarly, the minimum selling price of second gen PEF (that is, produced from agricultural waste and lignocellulosic feedstock) was estimated to be equal to 3.13 USD kg^−1^, with feedstock and utilities contributing significantly to the operating costs [24]. For all these reasons, (1R,3S)-(+)-camphoric acid (CA) (structure shown in Figure 1) is considered as a particularly interesting diacid to be used as monomer for the production of sustainable polyesters. In fact, CA can be obtained from the oxidation of D-(+)-camphor, a terpene known for its wide presence in nature (in greater abundance than its optical isomer), low price, and ease of production, which used to be produced in several thousands of tons per year for applications such as cosmetics, due to its characteristic fragrance, or as a plasticizer [28]. Specifically, D-(+)-camphor can be obtained from the steam distillation of the wood of *Cinnamomum camphora*, or *Camphora officinarum*, common trees growing in Southeast Asia and Africa, but now grown in many countries in the world [28,29,30,31]. CA is also known to be produced from turpentine, distilled from the resin of the tree *Pinus excelsa* [32,33]. In recent years, because of the renewed interest in biobased chemicals as a more sustainable alternative to petrochemical building blocks, research has been carried out on new sustainable processes for the production of CA [34] and on its utilization in new polymeric systems [35,36,37,38]. In particular, polymerization of low-molecular-weight homopolymers from CA and hexanediol (HD), as well as a variety of other diols, has been reported in the past in the literature [35]. Other monomers derived from camphor have also been used, such as rigid diols, to carry out random and multiblock copolyesters with PBS [39], and homopolymers with terephthalic acid or 2,5-FDCA [40]. Excellent functional properties for food packaging applications were found in polymeric systems containing CA, trans-1,4-cyclohexanedicarboxylate, and 1,4-butanediol [41], and also in random copolymers of CA, 2,5-FDCA and 1,4-butanediol [42]. Because of the steric hindrance caused by the methyl group in position 1 of the camphoric cycle, the polymerization of CA was found to be challenging, since a difference in reactivity between the two reactive groups of a difunctional monomer leads to a behavior which is similar to the one of a monofunctional chain terminator, preventing the achievement of high molecular weights. For this reason, the polymerization of CA had to be optimized to favor the reactivity of its sterically hindered acid moiety [41,42]. The same kind of optimization was carried out in this work, focusing on the random copolymerization of 2,5-FDCA with CA and HD. The best products that could be obtained used a feed of CA equal to 10, 20 and 25 mol% and were called PHFC09, PHFC15, and PHFC17, respectively. After being synthesized, they were purified and subjected to molecular, thermal, and structural characterization. Moreover, their properties for food packaging applications were assessed by means of mechanical and gas permeability properties.

## 2. Materials and Methods

### 2.1. Materials

A total of 98% purity 2,5-furandicarboxylic acid (2,5-FDCA) was purchased from Carbosynth Biosynth, (Compton, UK), 98% purity (1R,3S)-(+)-camphoric acid (CA) was purchased from J&K Scientific (Beijing, China), 97% purity 1,6-hexanediol (HD) was purchased from Tokyo Chemical Industry (Tokyo, Japan), and titanium (IV) tetrabutoxide (TBT) and titanium (IV) isopropoxide (TIP) were purchased from Sigma-Aldrich (Saint Louis, MO, USA). All reagents were used as purchased.

### 2.2. Synthesis

Homopolymeric poly(hexamethylene furanoate) (PHF) was synthesized from 2,5-FDCA (5.30 g, 34 mmol) and HD (11.9 g, 101 mmol, 200 mol% glycol excess compared to the diacid), while poly(hexamethylene furanoate-co-camphorate) random copolymers (PHFC) were synthesized in a one-pot process, from 2,5-FDCA (4.64 g, 30 mmol for PHFC09; 3.12 g, 20 mmol for PHFC15; 2.65 g, 17 mmol for PHFC17), CA (0.66 g, 3 mmol for PHFC09; 1.00 g, 5 mmol for PHFC15; 1.13 g, 6 mmol for PHFC17), and HD (19.5 g, 165 mmol for PHFC09; 14.77 g, 125 mmol for PHFC15; 13.39 g, 113 mmol for PHFC17; in all cases, 400 mol% glycol excess compared to the diacids). For all syntheses, 200 ppm of TBT and TIP were used as catalysts. All the reactions were carried out without the use of a solvent, in a 250 mL glass reactor placed inside a salt bath composed by a mixture of 50% KNO_3_, 30% NaNO_2_, and 20% NaNO_3_, with eutectic melting point equal to 140 °C. The process was a two-stage polymerization procedure typically found in the literature [3] for polyester synthesis. The first step was carried out under a nitrogen flow at a temperature set at 180 °C. Water was produced as the byproduct of the esterification reaction between the diacids and the glycol. It distilled from the reactor and was collected in a vacuum trap. After about 90 min for homopolymeric PHF and 120 min for PHFC copolymers, no more water could be collected, so a vacuum pump was connected to the vacuum trap, and vacuum was applied gradually to collect the excess glycol at first, then the glycol produced as the transesterification byproduct of the polymerization reaction between oligomers. During this second stage, the pressure was slowly decreased to 0.05 mbar (at a rate of about 20 and 5 bar min^−1^ for homopolymeric PHF and PHFC copolymers, respectively), and the temperature was slowly increased to 220 °C (at a rate of about 1 and 0.25 °C min^−1^ for homopolymeric PHF and PHFC copolymers, respectively), while the torque of the polymer mass was monitored. After about 150 min for homopolymeric PHF and 480 min for PHFC copolymers, no more glycol could be collected and the torque was found to have reached a plateau, so the reaction was interrupted and the final product was collected from the reactor.

### 2.3. Sample Preparation

The products were purified by dissolution in chloroform and precipitation in methanol, then dried overnight under the fume hood. After placing purified powder between two Teflon sheets, 2.5 g of it was compression-molded into films with a thickness of about 300 μm using a C12 laboratory press (Carver, Wabash, IN, USA). After melting the sample at a temperature 30 °C above its T_m_, a pressure of 9 ton m^−2^ was applied for 1 min. The compression-molded films had a diameter of at least 11 cm. They were stored in a desiccator for at least 14 days before being analyzed. The samples were stored in the desiccator for the entire duration of this study. The desiccator used silica gel as a drying agent containing methyl violet as an indicator. The effectiveness of the desiccator was tested before use by storing anhydrous calcium chloride and verifying that its weight difference before and after storing was lower than 0.1 mg. The desiccator was placed in a room where the temperature was kept in the 20–30 °C range throughout this study.

### 2.4. Molecular Characterization

Nuclear Magnetic Resonance Spectroscopy (NMR) was carried out with a 400 MHz Varian Inova instrument (Agilent Technologies, Palo Alto, CA, USA). For ^1^H-NMR, 10 mg of specimen was dissolved in 0.7 mL of deuterated chloroform, then analyzed in an NMR tube. For ^13^C-NMR, 40 mg of specimen was used.

Fourier Transform Infrared Spectroscopy (FTIR) experiments were performed at room temperature employing a Spectrum 3 Spectrometer (PerkinElmer, Waltham, MA, USA) with an ATR cell by the same producer.

### 2.5. Structural Characterization

Wide-angle X-ray scattering (WAXS) was carried out with an X’PertPro diffractometer (PANalytical, Almelo, The Netherlands) equipped with a solid-state X’Celerator detector. The analyses used X-rays from a copper source of wavelength equal to 0.154 nm, while the instrument moved at a rate of 100 s step^−1^, in 0.1° steps. After ignoring incoherent scattering, the degree of crystallinity (X_c_) was calculated by dividing the crystalline area (A_c_) by the total area of the diffraction curve (A_t_), where A_c_ was obtained by subtracting the amorphous area (A_a_) from A_t_. Scherrer equation, which ignores strain contribution to peak broadening, was employed for a rough estimation of crystallite size: L = Kλ (βcosθ)^−1^, where λ is the wavelength, β in radian is the full width at high medium (corrected for instrumental contribution) of the considered peak, θ is the Bragg angle (degree, 17°/2 in the present case), and K is the Scherrer constant, which depends on the crystal shape. Since in the present study the L value is used only to internal comparison between the samples, it was taken as K = 1.

### 2.6. Thermal Characterization

Differential Scanning Calorimetry (DSC) was carried out with a DSC6 (PerkinElmer, Waltham, MA, USA). A total of 5 mg of sample was heated under a flow of 20 mL min^−1^ of pure nitrogen from −30 to 190 °C at 20 °C min^−1^, cooled from 190 to −30 °C at 100 °C min^−1^, and finally heated again from −20 to 190 °C at 20 °C min^−1^. The glass transition temperature (T_g_) and the specific heat variation (ΔC_p_) were, respectively, determined as half the heat flow between the baselines before and after the glass transition, and from the height between the baselines. The melting temperature (T_m_) and the melting enthalpy (ΔH_m_) were, respectively, determined from the maximum and the total area of melting peaks. The same logic was applied to isotropizations (T_i_, ΔH_i_) and cold crystallizations (T_cc_, ΔH_cc_).

Thermogravimetric Analysis (TGA) was carried out with a TGA4000 (PerkinElmer, Waltham, MA, USA). A total of 10 mg of sample was heated under a flow of 40 mL min^−1^ of pure nitrogen from 40 to 800 °C at 10 °C min^−1^. T_5%_, T_onset_, and T_max_ were, respectively, determined as the temperature at which 5% weight loss is reached, weight loss begins, and derivative of the curve reaches its minimum.

### 2.7. Mechanical Tests

Tensile tests were carried out with an Instron 5966 (Instron, Norwood, MA, USA) using a transducer-coupled 1 kN load cell. Then, 5 mm × 50 mm film samples had their average thickness measured, then stretched at 10 mm min^−1^, from a gauge length of 2.0 cm. The results were expressed as stress–strain curves. The elongation at break (ε_b_) and the stress at break (σ_b_) were determined from the breaking point, and the same logic was applied to the yield elongation (ε_y_) and the yield stress (σ_y_). The elastic modulus (E) was determined by fitting the linear elastic range with a line with regression close to 0.99 and by calculating its slope.

### 2.8. Gas Permeability Tests

Gas permeability tests were carried out with a permeance testing device, type GDP-C (Brugger Feinmechanik GmbH, Munchen, Germany), equipped with an external thermostat HAAKE-Circulator DC10-K15 type (ThermoFisher Scientific, Waltham, MA, USA). The procedure followed the manometric method reported in [43,44,45]. Film samples with a diameter of at least 10 cm were placed between two chambers at controlled pressure, then vacuum was applied in one of the two, while the other was filled with pure O_2_ or CO_2_ (100 mL min^−1^), at 0% relative humidity and 23 °C. O_2_ and CO_2_ permeabilities were determined from the pressure vs. time plot using the typical equations found in the literature [46,47,48,49,50,51,52,53,54]. The membrane area used for the calculations was 78.4 cm^2^. The results were expressed in cm^3^ cm m^−2^ d^−1^ atm^−1^, where 1 cm^3^ cm m^−2^ d^−1^ atm^−1^ = 0.15228 Barrer. The instrument was calibrated in accordance with the manufacturer’s procedure (GDP-C Brugger Feinmechanik GmbH, Munich, Germany). Calibration was performed at 23 °C using a 50 μm steel blocking film with air as the test gas (sample area of 78.4 cm^2^), and at 23 °C with a 100 μm polyethylene terephthalate (PET) reference film under O_2_ gas flow (sample area of 78.4 cm^2^).

## 3. Results and Discussion

### 3.1. Synthesis and Molecular Characterization

Because of the steric hindrance caused by the methyl group in position 1 of the camphoric cycle, the polymerization of CA (Figure 1) had to be optimized to favor the reactivity of its sterically hindered acid moiety. Among the adjustments, the reaction time of the first step of the process was set to 120 min, longer than the 60–90 min necessary for this step in typical polyester synthesis. Another adjustment was the rate at which vacuum was applied: in fact, pressure was lowered at a rate of about 5 bar min^−1^, lower than the rate of about 20 bar min^−1^ used for the synthesis of homopolymeric PHF. Moreover, the glycol excess was set to 400 mol%, higher than the 100–200 mol% typically used for the synthesis of furan-based polyesters. All adjustments were meant to push the esterification equilibrium towards the oligomers. Further increasing the glycol excess was found to be counterproductive, since it decreased the viscosity of the oligomer, facilitating its loss when the excess glycol was removed by applying the vacuum, at the beginning of the second step.

**Figure 2:**
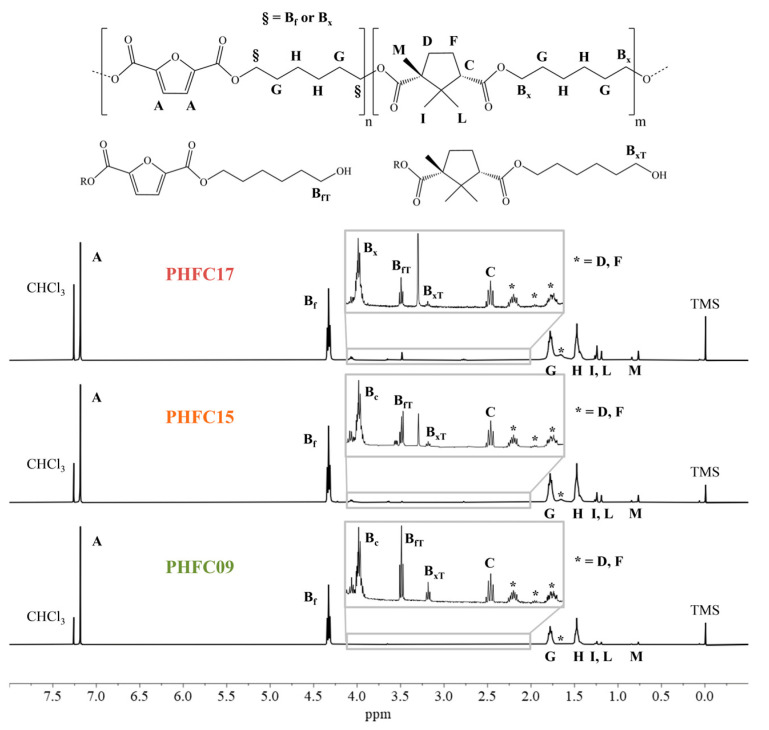
^1^H-NMR spectra of PHF, PHFC09, PHFC15, and PHFC17, with peak assignment. Insets: magnification in the 4.20–2.00 ppm range.

**Figure 3:**
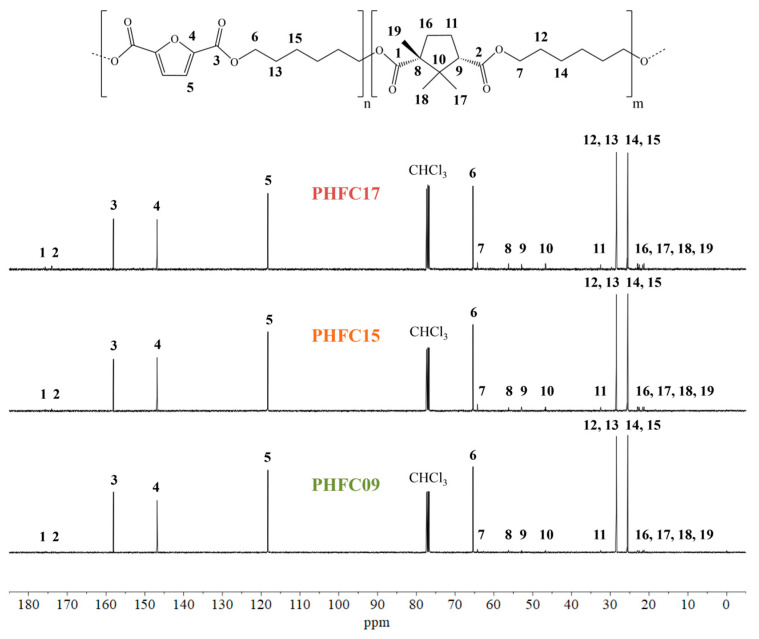
^13^C-NMR spectra of PHF, PHFC09, PHFC15, and PHFC17, with peak assignment.

The reaction products were purified, then their structure was confirmed by means of ^1^H-NMR and ^13^C-NMR analyses. The obtained spectra are shown in Figure 2 and Figure 3. Additional NMR and FTIR spectra of the monomers and polymers under study were also obtained and are shown in Figures S1 and S2. In all ^1^H-NMR spectra, the signal of non-deuterated impurities of deuterated chloroform can be observed as a singlet at 7.26 ppm, while in ^13^C-NMR, it is associated with a triplet centered at 77.03 ppm. In the samples of PHFC15 and PHFC17 used for ^1^H-NMR analyses, an unknown impurity originated a singlet at 3.48 ppm. Since the samples were purified, it is possible that the signal was originated by traces of methanol. In ^1^H-NMR spectra, singlet A was originated by the aromatic hydrogen atoms found in the furanic subunit. The furanic unit also originated glycolic multiplets B_f_ (4.33 ppm), G (1.66 ppm), and H (1.44 ppm). These signals were found to be multiplets because they were influenced by several possible combinations of furanic and camphoric subunits at the extremes of the glycol subunit. In particular, the glycolic methylene groups closer to the acid subunits originated the intense signal called B_f_ when they were located between two furanic subunits. These methylene groups were also influenced by the presence of a camphoric subunit and by the two different orientations of the camphoric subunit. There could be up to nine different combinations, each originating a different triplet, contributing to the complexity of multiplet B_x_ (4.06 ppm), which was hypothesized to account for all of them, apart from the one associated with peak B_f_. Peaks B_fT_ (3.65 ppm) and B_xT_ (3.39 ppm) were associated with the OH-terminated glycolic subunits having a furanic subunit and a camphoric subunit at the other end of the glycol, respectively. Triplet C (2.78 ppm) was identified as the lone hydrogen atom located in position α with respect to one of the two carboxylic moieties of the camphoric subunit. The methyl substituent located in position α to the other carboxylic moiety was identified with signal M (0.77 ppm). Aliphatic camphoric methylene groups D and F were associated with the four multiplets indicated with a “*” (at 2.55, 2.17, 1.78, 1.48 ppm), since each of their hydrogen atoms originated a different signal. Finally, the geminal camphoric methyl substituents originated signals I and L (1.25 and 1.19 ppm, respectively). Overall, the camphoric ^1^H-NMR signals were found to be comparable to the ones found for different camphoric copolymers in the literature [41,42]. In the case of ^13^C-NMR spectra, the attribution is shown in Figure 3. It is worth mentioning that glycolic peaks 6, 13, and 15 were primarily associated with methylene groups closer to furanic subunits, and they showed secondary peaks, respectively, called 7, 12, and 14, which should be associated with methylene groups closer to camphoric subunits. However, no peaks associated with other combinations were detected, possibly because their intensity was lower than the instrumental noise typical of ^13^C-NMR analyses. For this reason, the ^13^C-NMR spectra could not be used to calculate the degree of randomness of the random copolymers. The degree of randomness could also not be determined from ^1^H-NMR spectra, since the glycolic multiplets constituting signals B_x_, G, and H were highly superimposed and difficult to interpret. We can reasonably assume that polymers are random, having obtained them by polycondensation starting from monomers. Nevertheless, the molar percentage of camphoric subunits could be calculated for each polymer by dividing the normalized integral of ^1^H-NMR peak C with the sum of the normalized integrals of peaks A and C. These results are shown in Figure 2, together with the conversion of CA, defined as feed mol% divided by the mol% found by means of ^1^H-NMR. As qualitative confirmation of these results, it can be observed how, with increasing camphoric mol% in the reaction feed, camphoric ^1^H-NMR signals B_x_, C, D, F, I, L, and M grew in intensity compared to furanic signals A and B_f_. The same was true for camphoric ^13^C-NMR signals 1, 2, 7, 8, 9, 10, 11, 12, 14, 16, 17, 18, and 19, whose intensity increased compared to the one of furanic signals 3, 4, 5, 6, 13, and 15. In all cases, real copolymer composition was found to be very close to the feed one.

The polymers under study were also characterized by means of GPC analyses. The average number molecular weight (M_n_) and the polydispersity (Ð) values are shown in Table 1. The polydispersity values were consistently found to be close to two for all PHFC copolymers, as it is typical for polyesters synthesized with a two-step polycondensation process. This result shows good control over the degree of polymerization reached for all the polymeric chains and great reproducibility of the results. In terms of molecular weight, it is common knowledge that furan-based polyesters with an M_n_ higher than 20 kg mol^−1^ typically reach a plateau of functional performance [55]. After this threshold, increasing the molecular weight causes minor changes in the functional properties under examination [56]. In this work, the molecular weight of PHF was well above said threshold, while PHFC09 and PHFC15 were close to it, so the results of their functional characterization were considered comparable. Unlike them, PHFC17 had a molecular weight of about 13 kg mol^−1^, so its properties, particularly its functional properties, must have been influenced by it. As such, they were considered as not comparable to the ones of the other polymers under study.

**Figure 4:**
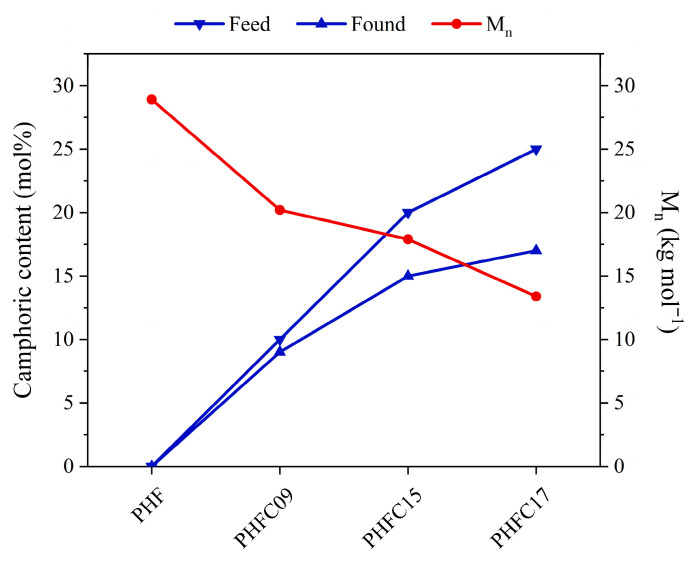
Camphoric content of the reaction feed and measured by means of ^1^H-NMR (left y axis), and number-average molecular weight measured by means of GPC (right y axis) for PHF, PHFC09, PHFC15, and PHFC17.

The feed and measured CA mol% are compared to the molecular weight of the PHFC copolymers in Table 1. The graph shows a clear trend of decreasing molecular weight with increasing percentages of CA feed, confirming the hypothesis that the poor reactivity of one of the two functional groups of CA led to a behavior in part similar to the one of a chain terminator. This effect also caused a lower conversion of CA into polymer, as shown by the growing difference between the feed and found mol% in Figure 4, with increasing feed CA mol%. Nevertheless, a conversion above 68 mol% was reached for all PHFC copolymers, and overall, the results show that good results can be obtained with the use of up to 20 mol% of CA in the reaction feed. As a final remark, it should be noted that inside the 4.20–2.00 ppm range of the ^1^H-NMR spectra (Figure 2), signals B_fT_ and B_xT_ might seem to show intensities with a trend, which contradicts the one observed with GPC analyses. This is only a perception due to the fact that the intensity of the signals inside the inset was normalized on the basis of signal B_x_. For this reason, for example, since the camphoric signals of PHFC09 are lower, the relative intensities of signals B_fT_ and B_xT_ appear higher.

### 3.2. Structural and Thermal Characterization

**Figure 5:**
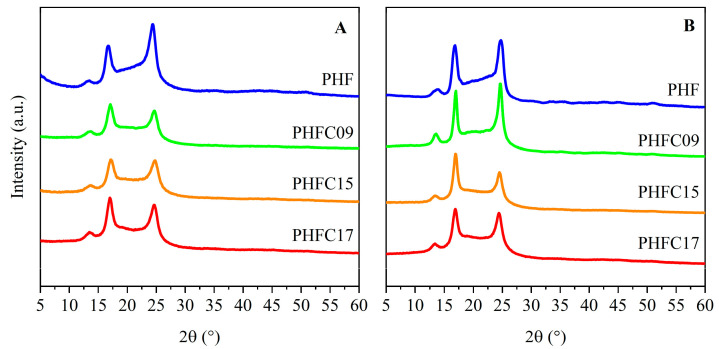
WAXS profiles of PHF, PHFC09, PHFC15, and PHFC17 samples: (**A**) powders; (**B**) compression-molded films.

In order to understand the structure of the PHFC copolymers and of their reference homopolymer, WAXS analyses were carried out. The results of the analyses are shown in Table 1 and Figure 5. As can be seen from the diffraction profiles, the main reflections of PHF were found at about 2θ = 13.4°, 17.0°, and 24.5°, both in purified powders and in compression-molded film samples, in accordance with what was reported in the literature [18]. PHFC copolymers had three reflections located at the same angles. These results indicate that all samples were semicrystalline, and it is likely that the crystalline domains of all samples were mainly constituted by PHF sequences. This finding is not surprising, since the camphoric subunits were found to be present at a percentage equal or lower than 17 mol%, and they are sterically bulkier and less symmetrical than furanic ones. The degree of crystallinity (Xc) for each sample was calculated and is reported in Table 1. The Xc of the PHFC copolymers was found to be in the 31–34% range for powders and films. In particular, films showed decreasing degree of crystallinity with increasing camphoric content. This is in accordance with what can be observed in Figure 1: at equal thickness of the film sample under observation, opacity decreased as camphoric content increased. Interestingly, the opacity of PHF was also observed in other studies for 2,4-PHF, an isomeric form of PHF based on 2,4-FDCA [57]. In that case, the opacity of the sample was interpreted as potentially correlated with the presence of a partially ordered 1D/2D phase, whose formation has been theorized for many 2,5-poly(alkylene furanoate) homopolymers [18]. The same correlation between degree of crystallinity and the camphoric content could not be observed for the powder samples: the variations observed could be due to variations in the drying conditions after purification. The crystal size (L) could be calculated with the Scherrer formula, and the results are in the 7–12 nm range. The crystal size of all film samples is slightly larger than that of the powder samples with the same composition.

**Figure 6:**
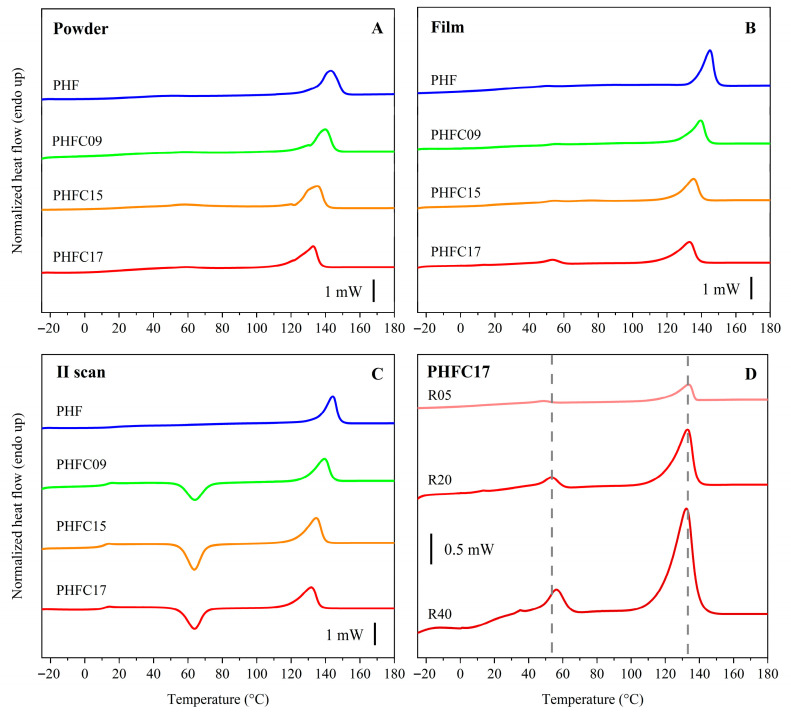
DSC curves of PHF, PHFC09, PHFC15, and PHFC17. (**A**) I DSC scan on powder samples. (**B**) I scan on film samples. (**C**) II scan on powder samples. (**D**) I scan at different heating rates expressed in °C min^−1^ on film samples of PHFC17.

The structure and the thermal transitions of PHFC copolymers could be further investigated with DSC analyses. The results can be observed in Table 2 and Figure 6. Homopolymeric PHF and PHFC copolymers were found to be rubbery and semicrystalline at room temperature, both in the form of purified powders and compression-molded films. In particular, in Figure 6A, the first scans on purified powder samples are shown. As can be observed, only one clear melting peak was found at 143 °C for PHF. The same melting peak was observed in PHFC copolymers, but its position shifted towards lower temperatures with increasing camphoric mol%: in fact, the melting temperature was equal to 140, 135, and 133 °C for PHFC09, PHFC15, and PHFC17, respectively. Exactly the same trend was observed for both the first and second heating scans of purified powders and compression-molded films. These observations were expected, since the high mol% of PHF units made it possible for PHF crystals to form in the random copolymers; however, the presence of camphoric units lead to increasingly less perfect crystals with a lower melting temperature. In accordance with the WAXS data, the melting enthalpy of PHFC copolymers also decreased compared to homopolymeric PHF (42 vs. about 33 J g^−1^ for PHF and PHFC copolymers), but no significant trend was observed among the copolymers. From the second heating scans, more evidence was gathered on the hypothesis that camphoric units interfered with the formation of crystallinity. In particular, the second heating scans on powder samples is shown in Figure 6C. As it can be observed, the neat melting enthalpy of homopolymeric PHF was found to be equal to 34 J g^−1^, while PHFC copolymers underwent a cold crystallization process, and their melting peak was mainly due to the crystallization developed during the heating scan. Specifically, PHFC09, PHFC15, and PHFC17 had a neat melting enthalpy of 5, 4, and 3 J g^−1^, respectively, confirming that the increasing mol% of camphoric units progressively lowered the ability to crystallize for the copolymers. The same phenomenon was observed for the second heating scans on compression-molded samples. This observation was also supported by the higher ΔC_p_ observed during the second heating scans, which was equal to 0.146 J g^−1^ °C^−1^ for homopolymeric PHF and increased to an approximate value of 0.400 J g^−1^ °C^−1^ for all PHFC copolymers. Interestingly, the formation of an additional endothermic peak was observed in compression-molded samples (Figure 6B). The endothermic enthalpy increased with increasing camphoric mol% in PHFC copolymers (1, 2, and 4 J g^−1^ for PHFC09, PHFC15, and PHFC17, respectively). Because of the known ability of furan-based polyesters to form mesomorph phases, experiments at different heating rates were carried out on the sample in which this peak was the most intense, PHFC17. The experiments are shown in Figure 6D. Mesomorph phases are partially ordered, and as such, they undergo isotropization, which is a first order transition but which, unlike melting transitions, is highly sensitive to changes in heating rate. In fact, it is known that partially ordered phases typically show a kinetically hindered isotropization while maintaining an enthalpic peak typical of first order transitions. Due to this behavior, mesomorph phase isotropization was also called “weakly first-order” in the literature [58]. The sensitivity to heating rate was observed in the case of Figure 6D, in which the melting peak at 133 °C melted at the same temperature regardless of the heating rate, while the isotropization peak shifted at 48, 54, and 56 for a heating rate of 5, 20, and 40 °C min^−1^, respectively, similarly to the position of the T_g_, which was found at 8, 12, and 20 °C at 5, 20, and 40 °C min^−1^, respectively. This result is evidence that the presence of a camphoric unit might have enhanced the formation of a partially ordered mesomorph phase in PHFC copolymers. Moreover, the literature suggests that this mesomorph phase might be smectic and not nematic, since it has been reported that smectic phases form predominantly in polyesters having glycolic subunits with an even number of methylene groups, and they have an isotropization temperature typically located between T_g_ and T_m_ [59,60,61].

**Figure 7:**
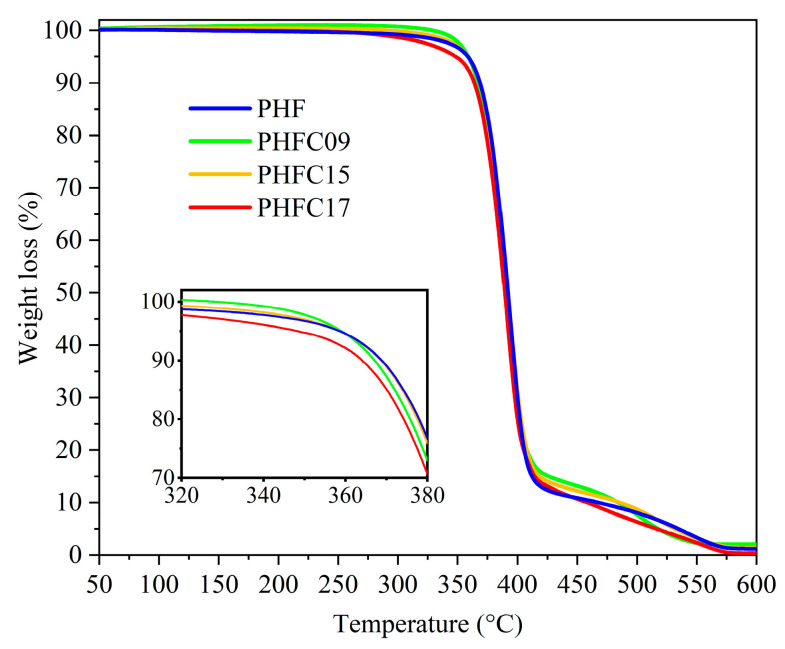
TGA curves of PHF, PHFC09, PHFC15, and PHFC17. Inset: magnification at low weight loss.

Finally, TGA experiments were carried out to determine the thermal stability of PHFC copolymers. The results are shown in Table 2 and Figure 7.

They were found to be comparable to the excellent ones of homopolymeric PHF, as expected from polymers with high aromatic content, thus ensuring a wide window of processability between the melting temperature and the degradation of PHFC copolymers. Specifically, the T_max_ and the T_onset_ were found to be approximately 395 °C and 372 °C, respectively. The T_5%_ of PHFC copolymers was also found to be comparable to homopolymeric PHF at a temperature of about 358 °C; however, PHFC17 showed a slightly lower T_5%_ value at 348 °C, possibly due to its lower molecular weight. As observed for similar furan-based polymers [18], after the main degradation step, the final part of the degradation process was found to have an intermediate step before the complete transformation of all residual weight into gas molecules.

### 3.3. Mechanical Tests

**Figure 8:**
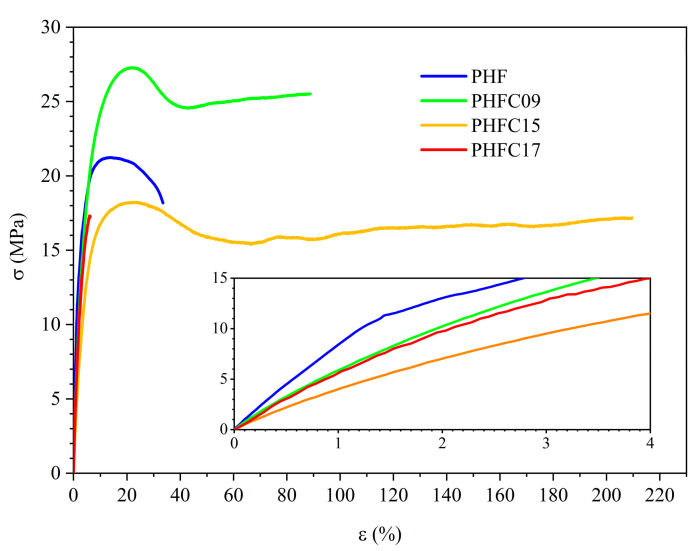
Stress–strain curves of PHF, PHFC09, PHFC15, and PHFC17. Inset: magnification at low elongation.

The results are shown in Table 3 and Figure 8. Homopolymeric PHF is relatively flexible, with a modulus equal to 906 MPa, and the elastic modulus (E) of PHFC copolymers was found to be lowered of about 300 MPa as a consequence of the copolymerization. This increase in flexibility can be attributed to the lower crystallinity and T_g_ observed in DSC experiments, due to the sterically hindered geometry of the camphoric subunits, which might have originated a plasticization effect. Due to the small variations in composition, no remarkable trend was observed for the elastic modulus of PHFC copolymers. Due to their semicrystalline structure, PHFC09 and PHFC25 showed clear necking with yield stress equal to 26 and 17 MPa, respectively, and yield strain equal to 21 and 18%, respectively. Because of its lower molecular weight, PHFC17 broke in the elastic range. Most interestingly, in spite of the lower elastic modulus, PHFC09 and PHFC15 showed much higher toughness than homopolymeric PHF, because of their higher elongation at break, equal to 93 and 223%, respectively. Toughness was estimated by calculating the area underneath the stress–strain curves, and for PHFC09 and PHFC15, it was found to, respectively, be 373 and 499% greater than that of homopolymeric PHF. These results were influenced by the difference in molecular weight of the copolymers, as previously discussed in Section 3.1. In particular, since the lower molecular weight caused by higher CA incorporation was found to cause a significant worsening of mechanical properties, PHFC17 represents the upper limit of CA incorporation using the synthetic method under study. However, the mechanical properties of PHFC09 and PHFC17 showed the great potential of camphoric acid to be used for its considerable toughening effect, in amounts as low as 9 and 15 mol%, for the production of flexible food packaging systems with high ductility and resistance to deformation.

### 3.4. Gas Permeability Tests

**Figure 9:**
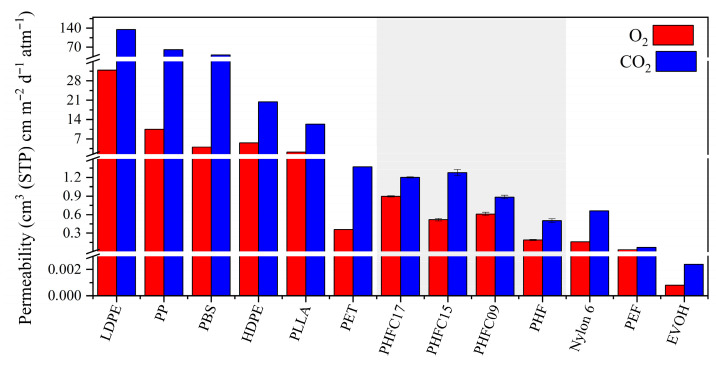
Gas permeability properties of PHF, PHFC09, PHFC15, and PHFC17 (highlighted in the gray area) compared to commercial LDPE [52], HDPE [52], PP [52], PET [62], Nylon 6 [52], PLLA (98% L) [52], PBS [63], PEF [64], and EVOH (32% ethylene) [52].

Gas permeability properties are fundamental in food packaging systems, since they provide protection against oxidation in fresh food or loss of carbonation in beverages, thus increasing their shelf life and reducing food loss and food waste. The gas permeability properties of PHF, PHFC09, PHFC15, and PHFC17 are shown in Table 3, while in Figure 9, they are compared to several petrochemical and biobased commercial polymers. The breaks in the y axis divide these polymers in four classes. The first range, between the orders of magnitude of 101 and 102 cm^3^(STP) cm m^−2^ d^−1^ atm^−1^, includes the CO_2_ permeability of low density poly(ethylene) (LDPE) [52], poly(propylene) (PP) [52], and biobased poly(butylene succinate) (PBS) [53]. These permeabilities are too high to be suitable for the production of bottles for carbonated beverages. The second class, between the orders of magnitude of 100 and 101 cm^3^ (STP) cm m^−2^ d^−1^ atm^−1^, includes the O_2_ permeability of LDPE [52], PP [52], and PBS [53], and both O_2_ and CO_2_ permeabilities of high density poly(ethylene) (HDPE) [52] and biobased poly(lactic acid) (PLLA) [52]. These permeabilities can be suitable for the production of food packaging meant for foodstuff, which requires low grade protection from oxidation or loss of carbonation. The third class, between the orders of magnitude of 10^−2^ and 100 cm^3^ (STP) cm m^−2^ d^−1^ atm^−1^, includes the permeabilities of poly(ethylene terephthalate) (PET) [54], Nylon 6 [52], and biobased poly(ethylene furanoate) (PEF) [55]. These permeabilities are excellent for the production of food packaging meant to increase the shelf life of the packaged product. Finally, the fourth class, in the order of magnitude of 10^−3^ cm^3^ (STP) cm m^−2^ d^−1^ atm^−1^, includes the permeabilities of few specialty polymers such as poly(ethylene vinyl alcohol) (EVOH) [52], which are almost impermeable to gases and are often used in multicomponent (thus less easily recyclable) food packaging systems, paired with external layers of polymers with higher mechanical properties and greater resistance to humidity, such as polyolefins. Homopolymeric PHF and PHFC copolymers belong to the third class of polymers, and the copolymers showed permeability only slightly higher than the ones of PHF. Within these three copolymers, no particular trend of O_2_ or CO_2_ permeability was observed on the basis of the camphoric mol%, and the minor variations observed between samples could be attributed to the statistical error of the instrument used. The slightly worse gas permeability properties on PHFC copolymers compared to PHF could be attributed to two factors: as discussed earlier, the T_g_ of all PHFC copolymers was found to be lower than that of homopolymeric PHF. This might have implied a greater amount of free volume in PHFC copolymers, leading to an easier passage of gas molecules through the material. Slightly worse permeability values could also be due to the presence of a mesomorph phase found in PHFC copolymers. Mesophases have been found in the literature to be responsible for excellent gas permeability properties in amorphous and rubbery furan-based systems, such as PPeF [56,57]. However, in semicrystalline polymers, the simultaneous presence of an amorphous, crystalline, and mesomorph phase has been hypothesized to cause a greater number of so called disclinations, which are interfaces of different phases and facilitate the passage of gas through the material [58,59,60], as it was observed in the case of amorphous and semicrystalline 2,4-PBF [61]. In any case, all polymers under study showcased excellent gas permeability properties, comparable to the ones of commonly used PET. Interestingly, the lower molecular weight of PHFC17 did not seem to have caused significant pejorative effects on the gas permeability properties of the copolymer, compared to the other copolymers under study.

## 4. Conclusions

In recent years, environmental and institutional pressures have been pushing innovation on polymeric materials and on food packaging towards the utilization of new renewable resources. In the field of polyester synthesis, camphoric acid (CA) is an interesting biobased diacid derived from camphor, one of the most widespread among all commercial fragrances. This work had the objective of combining CA, 2,5-FDCA and biobased HD to synthesize random copolymers. The optimization of the synthesis allowed to prepare three random copolymers successfully, called PHFC09, PHFC15, and PHFC17, with 9, 15, and 17 mol% of camphoric acid, respectively. The purified powders and compression-molded films were subjected to characterization. The most important findings are as follows:

- The mechanical properties were significantly improved compared to the homopolymer of reference, PHF. In particular, in the case of PHFC15, flexibility improved of about 33%, elongation at break by 430%, and toughness by 223%.
- The thermal stability and the gas permeability properties did not change significantly compared to PHF. In particular, the gas permeability properties were excellent, comparable to the ones of commercial PET and superior than those of polyolefins and other biobased polymeric systems, such as PLLA and PBS.
- The presence of camphoric acid units favored the formation of a mesomorph phase in all PHFC copolymers, and it was hypothesized that its structure could be smectic.

Among the copolymers under study, PHFC15 was found to be the best compromise between molecular weight and functional properties, with a number average molecular weight higher than 20 kg mol^−1^ and a toughness higher than 200 J m^−3^. Overall, the use of camphoric acid in the polymerization process made it possible to utilize a lower percentage of 2,5-FDCA, thus potentially reducing the costs of production of furan-based polyesters. Within the framework of the sustainable development model of the circular economy, this work highlighted the potential of PHFC copolymers for the production of cost-efficient, flexible, and biobased sustainable food packaging, with gas permeability properties comparable to the ones of its reference homopolymer, PHF, and with greatly improved mechanical properties.

## Figures and Tables

**Table 1:** ^1^H-NMR, GPC, and WAXS data on PHF, PHFC09, PHFC15, and PHFC17.

Physical State	C Feed (mol%)	C Found ^1^ (mol%)	**χ**^1,2^ (%)	M_n_ ^3^ (g mol^−1^)	Đ ^3^	X_c_ ^4^ (%)	FWHM ^4^ (°)	L ^4^ (nm)
PHF								
Powder	0	0	-	28,900	2.3	37	1.1	8
Film						35	1.0	9
PHFC09								
Powder	10	9	90	20,200	1.9	33	1.1	8
Film						34	0.7	12
PHFC15								
Powder	20	15	75	17,900	2.0	31	1.2	7
Film						32	0.8	11
PHFC17								
Powder	25	17	68	13,400	2.0	34	1.1	8
Film						31	0.9	9

^1^ Measured by means of ^1^H-NMR. ^2^ Conversion of CA. ^3^ Measured by means of GPC. ^4^ Measured by means of WAXS. Full width at half-maximum of peak at 2θ = 17°. L calculated with Scherrer formula.

**Table 2:** DSC and TGA data of PHF, PHFC09, PHFC15, and PHFC17. Data on film samples was obtained from films rested for 18 days. All DSC data from first scans, unless otherwise specified. DSC data on second scans are from film samples, identical to second scans on powder samples.

Sample		T_g_ (°C) ^1^ ΔC_p_ (J g^−1^ °C^−1^) ^1^	T_i_ (°C) ^1^ ΔH_i_ (J g^−1^) ^1^	T_cc_ (°C) ^1^ ΔH_cc_ (J g^−1^) ^1^	T_m_ (°C) ^1^ ΔH_m_ (J g^−1^) ^1^	T_5%_^2^	T_onset_^2^	T_max_^2^
PHF								
Powder		23	-	-	143	358	372	395
		0.210	-	-	42			
Film		22	-	-	145			
		0.208	-	-	38			
II scan		18	-	-	144			
		0.146	-	-	34			
PHFC09								
Powder		21	-	-	140	359	369	392
		0.244	-	-	33			
Film		20	55	-	140			
		0.202	1	-	34			
II scan		11	-	64	139			
		0.451	-	26	31			
PHFC15								
Powder		21	-	-	135	359	371	395
		0.304	-	-	35			
Film		19	54	-	135			
		0.174	2	-	33			
II scan		10	-	64	134			
		0.499	-	33	37			
PHFC17								
Powder		18	-	-	133	348	370	391
		0.381		-	33			
Film	R05	8	48	-	133			
		0.114	3	-	37			
	R20	12	54	-	133			
		0.148	4	-	36			
	R40	20	56	-	133			
		0.377	6	-	39			
II scan		10	-	64	132			
		0.364	-	29	32			

^1^ Measured by means of DSC. ^2^ Measured by means of TGA.

**Table 3:** Mechanical and gas permeability properties of PHF, PHFC09, PHFC15, and PHFC17. O_2_ and CO_2_ permeabilities measured at 0% relative humidity, 23 °C, and expressed in cm^3^ (STP) cm m^−2^ d^−1^ atm^−1^.

Property	PHF	PHFC09	PHFC15	PHFC17
Thickness (μm)	360 ± 10	237 ± 8	178 ± 9	263 ± 6
O_2_ Permeability	0.19 ± 0.01	0.61 ± 0.03	0.52 ± 0.02	0.90 ± 0.01
CO_2_ Permeability	0.50 ± 0.03	0.88 ± 0.03	1.28 ± 0.05	1.20 ± 0.01
E (MPa)	910 ± 30	620 ± 90	520 ± 70	600 ± 40
σ_y_ (MPa)	-	26 ± 2	17 ± 1	-
ε_y_ (%)	-	21 ± 3	18 ± 4	-
σ_b_ (MPa)	22 ± 1	24 ± 1	17 ± 1	17 ± 2
ε_b_ (%)	42 ± 4	90 ± 20	220 ± 30	6 ± 1
Toughness (J m^−3^)	6 ± 1	27 ± 4	34 ± 5	0.9 ± 0.2

## Data Availability

The original contributions presented in this study are included in the article. Further inquiries can be directed to the corresponding author.

## Supplementary Materials

The following supporting information can be downloaded at https://www.mdpi.com/article/10.3390/polym18020255/s1. Figure S1: ^1^H-NMR spectra of the monomers used as reagents for this study; Figure S2: FTIR spectra of CA and of the polymers under study.
