# Supplementary material for: Biobased Random Copolymers of Poly(Hexamethylene Furanoate) for Sustainable Food Packaging: Camphoric Acid as a Valuable Co-Monomer for Improved Mechanical Properties
Source: Polymers (Basel). 2026 Jan 17;18(2):255. doi: 10.3390/polym18020255 (PMC12846005; doi:10.3390/polym18020255)
Supplement: Supplementary file 1 [file polymers-18-00255-s001.zip › polymers-4094638-supplementary.pdf]

Supplementary Information

for

Biobased random copolymers of poly(hexamethylene furanoate) for sustainable food packaging: camphoric acid as a valua-ble co-monomer for improved mechanical properties

Enrico Bianchi[1], Michelina Soccio[1,2], Valentina Siracusa[3], Massimo Gazzano[4] and Nadia Lotti[1,2,5,]*

[1] Department of Civil, Chemical, Environmental and Materials Engineering, University of Bologna, Via Terracini 28, 40131 Bologna, Italy

[2] Interdepartmental Center for Industrial Research on Advanced Applications in Mechanical Engineering and Materials Technology, CIRI-MAM, University of Bologna, Bologna, Italy

[3] Department of Chemical Science, University of Catania, Viale A. Doria 6, Catania 95125, Italy

4 Synthesis and Photoreactivity Institute, CNR, Via Gobetti 101, 40129 Bologna, Italy

[5] Interdepartmental Center for Agro-Food Research, CIRI-AGRO, University of Bologna, Bologna, Italy

*Correspondence: nadia.lotti@unibo.it

2 Pages

2 Figures

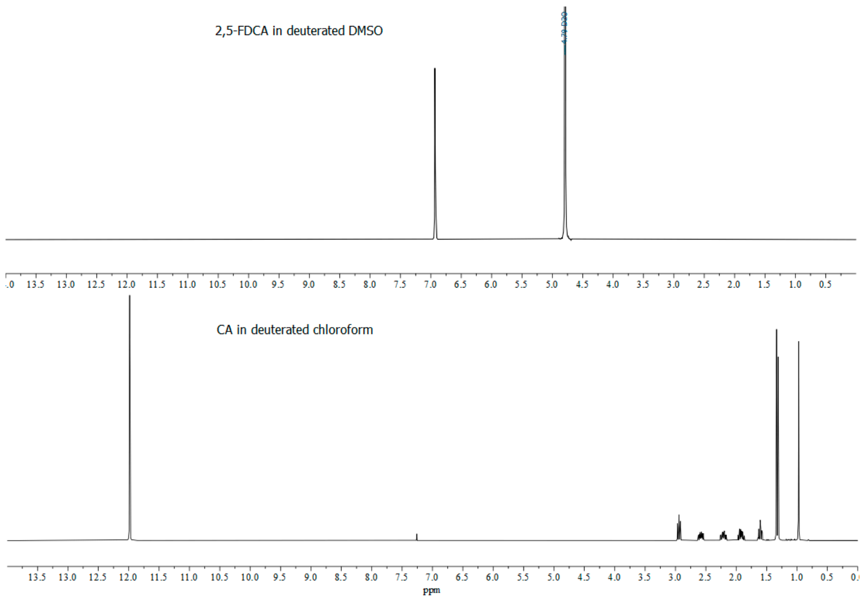

Figure S1. ^{1}H-NMR spectra of the monomers used as reagents for this study.

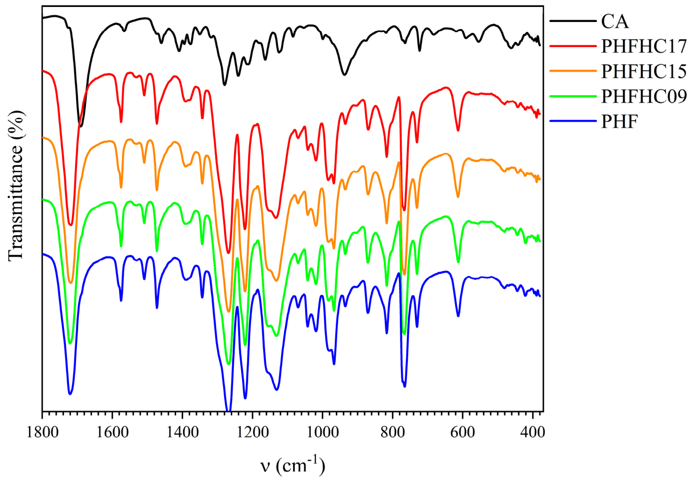

Figure S2. FTIR spectra of CA and of the polymers under study.
